# Categorical Dimensions of Human Odor Descriptor Space Revealed by Non-Negative Matrix Factorization

**DOI:** 10.1371/journal.pone.0073289

**Published:** 2013-09-18

**Authors:** Jason B. Castro, Arvind Ramanathan, Chakra S. Chennubhotla

**Affiliations:** 1 Department of Psychology, Bates College, Lewiston, Maine, United States of America; 2 Program in Neuroscience, Bates College, Lewiston, Maine, United States of America; 3 Computational Data Analytics Group, Computational Science and Engineering Division, Oak Ridge National Laboratory, Oak Ridge, Tennessee, United States of America; 4 Department of Computational and Systems Biology, University of Pittsburgh, Pittsburgh, Pennsylvania, United States of America; MPI f. med. Research, Germany

## Abstract

In contrast to most other sensory modalities, the basic perceptual dimensions of olfaction remain unclear. Here, we use non-negative matrix factorization (NMF) – a dimensionality reduction technique – to uncover structure in a panel of odor profiles, with each odor defined as a point in multi-dimensional descriptor space. The properties of NMF are favorable for the analysis of such lexical and perceptual data, and lead to a high-dimensional account of odor space. We further provide evidence that odor dimensions apply categorically. That is, odor space is not occupied homogenously, but rather in a discrete and intrinsically clustered manner. We discuss the potential implications of these results for the neural coding of odors, as well as for developing classifiers on larger datasets that may be useful for predicting perceptual qualities from chemical structures.

## Introduction

Our understanding of a sensory modality is marked, in part, by our ability to explain its characteristic perceptual qualities [1], [2]. To take the familiar example of vision, we know that the experience of color depends on the wavelength of light, and we have principled ways of referring to distances between percepts such as ‘red’, ‘yellow’ and ‘blue’ [2], [3]. In olfaction, by contrast, we lack a complete understanding of how odor perceptual space is organized. Indeed, it is still unclear whether olfaction even *has* fundamental perceptual axes that correspond to basic stimulus features.

Early efforts to systematically characterize odor space focused on identifying small numbers of perceptual primaries, which, when taken as a set, were hypothesized to span the full range of possible olfactory experiences [4]–[6]. Parallel work applied multidimensional scaling to odor discrimination data to derive a two-dimensional representation of odor space [7], [8], and recent studies using dimensionality reduction techniques such as Principal Components Analysis (PCA) on odor profiling data have affirmed these low-dimensional models of human olfactory perception [9]–[11]. A consistent finding of these latter studies is that odor percepts smoothly occupy a low dimensional manifold whose principal axis corresponds to hedonic valence, or “pleasantness”. Indeed, the primacy of pleasantness in olfactory experience may be reflected in the receptor topography of the olfactory epithelium [12] as well as in early central brain representations [13].

Here, we were interested in explicitly retaining additional degrees of freedom to describe olfactory percepts. Motivated by studies suggesting the existence of discrete perceptual clusters in olfaction [14], [15] we asked whether odor space is amenable to a description in terms of sparse perceptual dimensions that apply categorically. To do so, we applied non-negative matrix factorization (NMF) [16]–[19] to the odor profile database compiled by Dravnieks [20] and analyzed in a number of recent studies [9]–[11]. NMF and PCA are similar in that both methods attempt to capture the potentially low-dimensional structure of a data set; they differ, however, in the conditions that drive dimensionality reduction. Whereas basis vectors obtained from PCA are chosen to maximize variance, those obtained from NMF are constrained to be non-negative. This constraint has proven especially useful in the analysis of documents and other semantic data where data are intrinsically non-negative [19], [21] – a condition that is met by the Dravnieks database.

Applying NMF, we derive a 10-dimensional representation of odor perceptual space, with each dimension characterized by only a handful of positive valued semantic descriptors. Odor profiles tended to be categorically defined by their membership in a single one of these dimensions, which readily allowed co-clustering of odor features and odors. While the analysis of larger odor profile databases will be needed to generalize these results, the techniques described herein provide a conceptual and quantitative framework for investigating the potential mapping between chemicals and their corresponding odor percepts.

## Materials and Methods

### Non-Negative Matrix Factorization (NMF)

Non-negative matrix factorization (NMF) is a technique proposed for deriving low-rank approximations of the kind [16]–[18]:
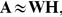
(1)where 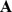 is a matrix of size 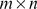 with non-negative entries, and 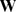 and 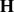 are low-dimensional, non-negative matrices of sizes 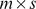 and 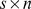 respectively, with 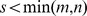. The matrices 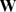 and 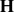 represent feature vectors and their weightings. NMF has been widely used for its ability to extract perceptually meaningful features, from high dimensional datasets, that are highly relevant to recognition and classification tasks in several different application domains.

To derive 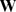 and 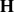 we used the alternate least squares algorithm originally proposed by Paatero [17]. Realizing that the optimization problem is convex in either 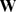 and 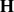, but not both, the algorithm iterates over the following steps:

1. assume 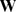 is known and solve the least squares problem for 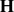 using:

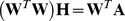

1. set negative elements of 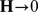

2. assume 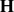 is known and solve the least squares problem for 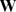 using

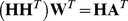

1. set negative elements of 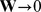.

We used the standard implementation of non-negative factorization algorithm ( nnmf.m) in Matlab (Mathworks, Inc.). Given the size of the odor profile matrix (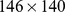), the speed of convergence was not an issue. As a stopping criterion, we chose a value of 1000 for the maximum number of iterations. Given the iterative nature of the algorithm and small size of the dataset, we expect the algorithm to reach a global minimum for small 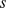 and a fixed point for large 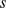.

Note that a minimum solution obtained by matrices 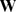 and 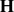 can also be satisifed by the pairs such as 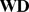 and 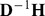 for any nonnegative 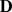 and 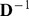. Thus, scaling and permutation can cause uniqueness problems, and hence the optimization algorithm typically enforces either row or column normalization in each iteration of the procedure outlined above.

### Cross-validation procedure with training and testing sets

The choice of sub-space dimension 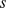 is problem dependent. Our strategy was to iterate over the sub-space dimension from 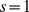 to 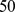, dividing the data matrix 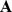 each time into random but equal-sized training and testing halves. We kept track of the residual error in the form of the Frobenius error norm: 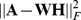 for both training and testing sets. For each choice of 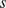 we repeated this division 250 times, with a stopping criterion of 1000 iterations, to report the statistics on residual errors. In addition, once an optimal sub-space dimension is chosen, we report the most stable version of the basis matrix, by computing KL-divergence between every pair of the 250 instances of 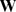 from the training set and picking 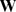 with the lowest mean KL-divergence value.

### Scrambling odor profiles

We applied NMF to scrambled perceptual data, that is elements of A are scrambled (randomly reorganized) before analyzing with NMF. Three different scrambling procedure were implemented. First was odorant shuffling where the column values of A are randomly permuted in each row. The second was descriptor shuffling where the row values of matrix A are randomly permuted in each column. Finally, we scrambled the elements of the entire matrix, that is indiscriminate shuffling of both descriptors and odorants entries.

### Consensus matrix

We tested the stability of the NMF results on the original and scrambled versions of the perceptual data using a consensus clustering algorithm proposed in [22], [23]. Because NMF is an iterative optimization algorithm, it may not converge to the same solution each time it is run (with random initial conditions). For a sub-space of dimension 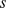, NMF algorithm groups descriptors and odorants into 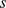 different clusters. If the clustering into 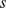 classes is strong, we expect the assignment of descriptors or odorants to their respective clusters will change only slightly from one run to another. We quantified this with a consensus matrix. For illustration, we will work with cluster assignments made to the descriptors. In particular, each descriptor 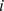 is assigned to a meta-descriptor 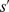, where 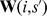 is the highest among all the values of 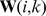 with 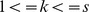.

We first initiated a zero-valued connectivity matrix 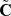 of size 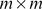. For each run of NMF, we updated the entries of the connectivity matrix by 1, that is 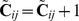 if descriptors 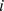 and 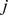 belong to the same cluster, or 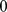 if they belong to different clusters. Averaging the connectivity matrix over all the runs of NMF gives the consensus matrix 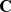, where the maximum value of 1 indicates that descriptors 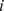 and 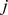 are always assigned to the same cluster. We ran NMF for 250 runs to ensure stability of the consensus matrix. If the clustering is stable, we expect the values in 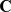 to be close to either 0 or 1. To see the cluster boundaries, we can use off-diagonal elements of 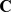 as a measure of similarity among descriptors, and invoke an agglomerative clustering method where one starts by assigning each descriptor to its own cluster and then recursively merges two or more most similar clusters until a stopping criterion is fulfilled. The output from the agglomerative clustering method can be used to reorder the rows and columns of 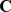 and make the cluster boundaries explicit.

### Cophenetic correlation coefficient

We then evaluated the stability of the clustering induced by a given sub-space dimension 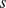. While visual inspection of the reordered 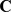 can provide qualitative insights into the stablity of cluster boundaries, we seek a quantitative measure by using the cophenetic correlation coefficient approach suggested in [23]. Note that there are two *distance* matrices to work with. The first distance matrix is induced by the consensus matrix generated by 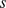-dim NMF decomposition. In particular, the distance between two descriptors is taken to be 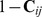. The second distance matrix is one induced by a agglomerative clustering method, such as the average linkage hierarchical clustering (HC). In particular the off-diagonal elements of the consensus matrix can be used as distance values to generate hierarchical clustering (HC) of the data (in Matlab, invoke: linkage.m with average linkage option). HC imposes a tree structure on the data, even if the data does not have a tree-like dependencies and is also sensitive to the distance metric in use. HC generates a dendrogram and the height 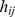 of the tree at which two elements are merged provide for the elements of the second distance matrix. The cophenetic correlation coefficient 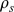 is defined to be the Pearson correlation value between the two distance matrices. If the consensus matrix is perfect, with elements being either 0 or 1, then 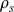 is 1. When the consensus matrix elements are between 0 and 1, then 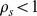.

We plot 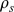 vs 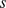 for increasing values of 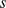. The results of such analyses are in some cases helpful for choosing an optimal subspace size. If a given clustering (say, for subspace size of 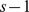) is highly reliable across repeated factorizations (that is, the same sets of descriptors and the same sets of odors tend to co-cluster), and hence 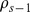 is very high, then one is motivated to retain at least (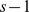) dimensions. If increasing this subspace size (to 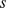, 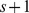, etc) leads to systematically less reliable clustering, 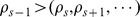, one is motivated to retain the more conservative estimate of dimensionality (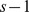). That said, we note that cophenetic correlation analyses can often provide better grounds for excluding certain choices of subspace size that lead to unreliable clustering, rather than privileging a specific number as ‘the’ dimensionality of the data. Note we seek solutions where 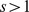 because for 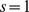 the correlation coefficient 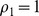. We also performed a similar consensus clustering and cophenetic coefficient analysis in the odorant space using the entries in 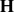.

### Odor space visualization

We use a variant of stochastic neighbor embedding method [24], [25] to visualize the high-dimensional odor space organized by NMF. In particular, we first generated the consensus matrices for clustering descriptors and odorants, and used them separately as similarity matrices in the stochastic neighbor embedding algorithm. We used the code from http://homepage.tudelft.nl/19j49/t-SNE.html and ran it with default parameters.

## Results

### Dimensionality of odor space

We analyzed the published data set of Dravnieks [20], which catalogs perceptual characteristics of 144 monomolecular odors. Each odor in this data set is represented as a 146 dimensional vector (an odor profile), with each dimension corresponding to the rated applicability of a given semantic label, such as ‘sweet’, ‘floral’, or ‘heavy’. Because these are strictly non-negative quantities (i.e. a given semantic label either applies, or does not), we reasoned this could be meaningfully exploited when reducing the dimensionality of profiling data. Thus, we applied NMF to the profiling data in an effort to obtain a perceptual basis set corresponding to ‘parts’ or ‘features’, as has been observed in the analysis of images [18] and text [18], [21].

**Figure 1:**
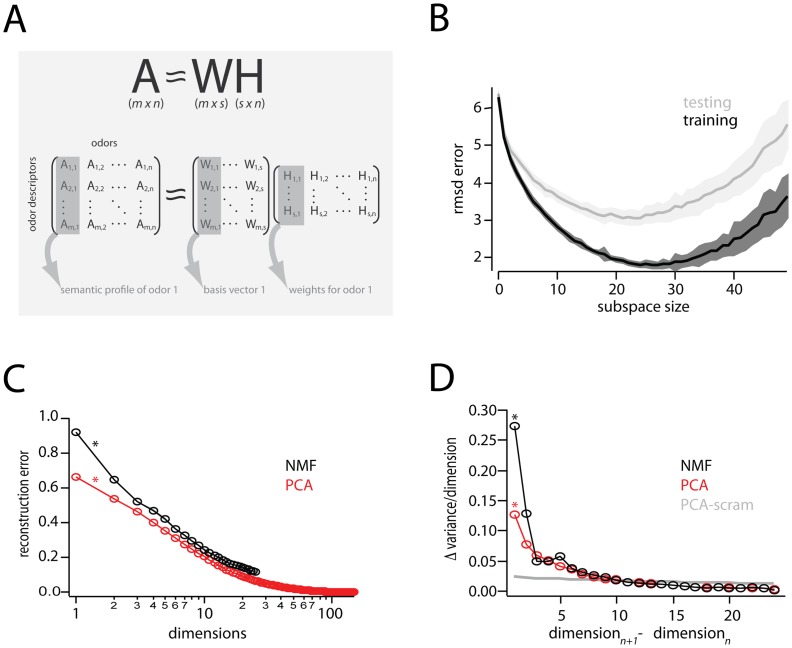
Summary of non-negative matrix factorization (NMF) applied to odor profiling data. 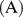
 Schematic Overview: NMF seeks a lower, *s*-dimensional approximation of a matrix 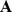 as the product of matrices 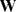 and 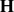. 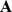 is 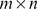, consisting in the present study of 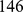 odor descriptors 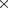 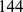 odors. A given column of 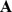 is the semantic profile of one odor, with each entry providing the percent-used value (see methods) of a given descriptor. Columns of 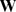 are basis vectors of the reduced, s-dimensional odor descriptor space. Columns of 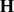 are 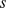-dimensional representations (weights) of the odors in the new basis. 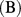 Plot of residual error between perceptual data, 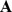, and different NMF-derived approximations. 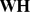. For each choice of subspace, data were divided into random training and testing halves, and residual error between 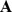 and 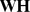 computed. One-hundred such divisions into training and testing were used to compute the standard errors shown (shaded areas). 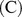 Reconstruction error (fraction of *unexplained* variance) for PCA and NMF vs. number of dimensions. The change in reconstruction error for the first interval is indicated by asterisks(*), and corresponds to the first point in the next panel. 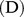 Change in reconstruction error for PCA and NMF, compared to the change in reconstruction error for PCA performed on a scrambled matrix (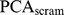). 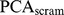 is used to estimate the cutoff number of dimensions for which a given dimensionality reduction method is explaining only noise in a dataset. Note that each point, 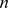, is actually the difference in reconstruction error between dimensions 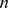 and 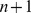 (by way of illustration, points with an asterisk in this panel denote corresponding intervals in the previous panel 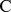).

NMF seeks a low-rank approximation of a matrix 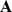 (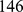 descriptors 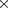 144 odors in the present case) as the product 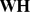, where the 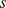 columns of 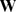 are non-negative basis vectors (146-D vectors of odor descriptors in the present case), and the columns of 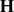 are the new 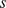-dimensional representations of the original odors (144 columns, in the present case) ( Fig. 1A). Figure 1B shows the root-mean-squared (RMS) residual (see Methods) between 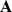 and its approximation 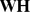 for subspaces ranging from 1 to 50 (100 equal divisions of 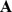 into training and testing subsets, for each choice of subspace). The residual attained a minimum for a subspace choice of 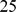, and increased for larger subspaces. In addition, the width of the error bars increased on the training and testing residuals after subspace 25. Increasing the number of iterations used for training the NMF model only marginally reduced the size of the error bars. We speculate that the energy landscape is becoming increasingly rugged, with the existence of many more local minima to potentially trap the learning of NMF model parameters. In particular, NMF employs a non-linear optimization method, and hence it is possible that the each time the method is run, it finds a local minimum that is different and far away from a global minimum. Hence, the error bars on the residuals are large and continue to increase with increasing subspace dimensionality 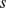 because of the ruggedness in the landscape and the limited size of odor profile data used for training the model.

**Figure 2:**
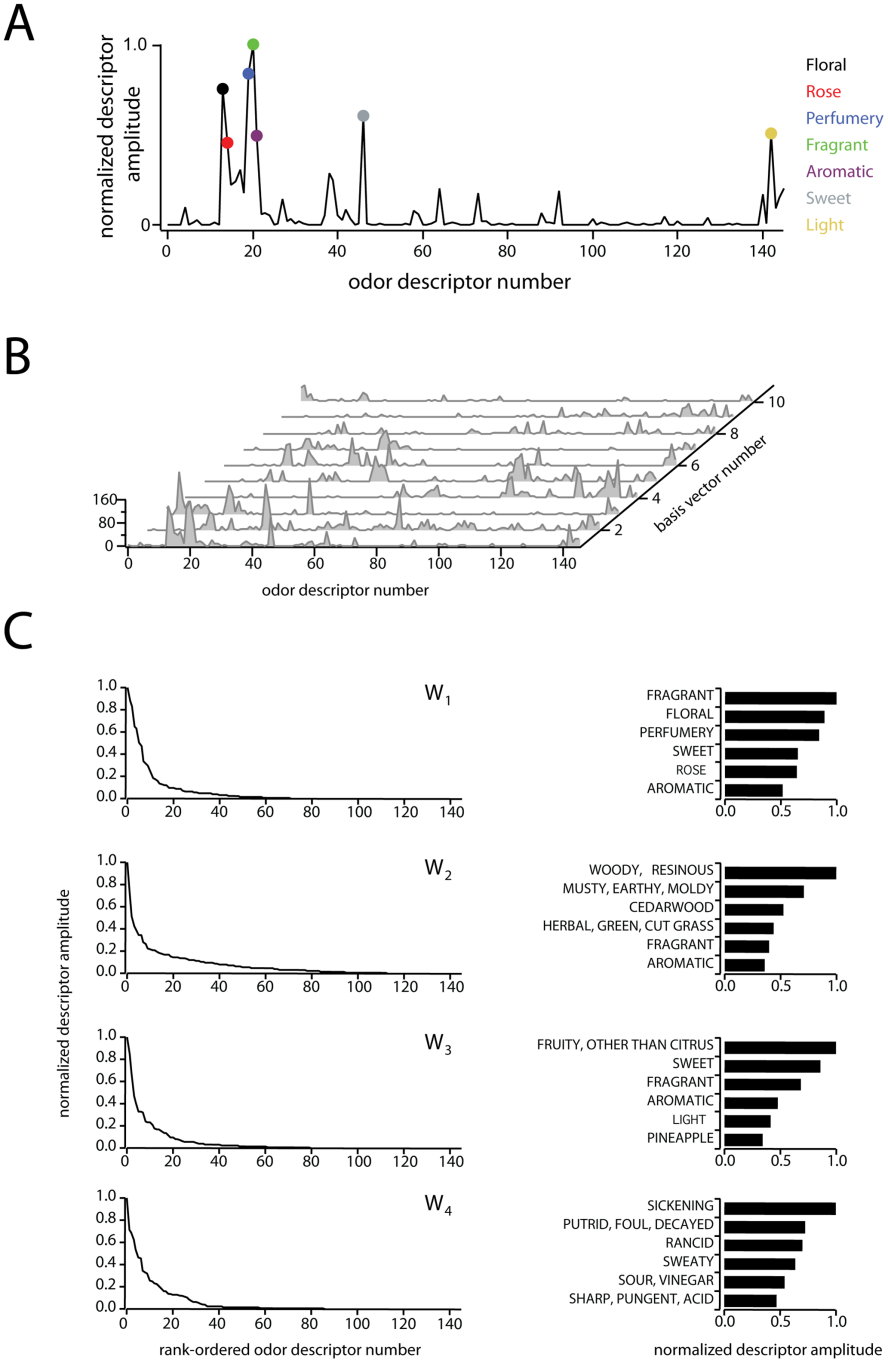
Properties of the perceptual basis set 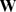. Plot of normalized odor descriptor amplitude vs. odor descriptor number for the basis vector 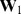. Each point along the x-axis corresponds to a single odor descriptor, and the amplitude of each descriptor indicates the descriptor's relevance to the shown perceptual basis vector. Colored circles show the 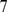 largest points in the basis vector, and descriptors corresponding to these points are listed to the right. 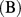 Waterfall plot of the 10 basis vectors constituting 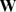, used in subsequent analyses. Note that each vector contains many values close to or equal to zero. 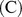 Detailed view of the first four basis vectors and their leading values. Left column: peak-normalized, rank ordered basis vectors, illustrating their sparseness and non-negativity. Right column: semantic descriptors characterizing the first four basis vectors. Bars show the first six rank-ordered, peak-normalized components of basis vectors 1 through 4 (subset of data from left column). The semantic label for each component is show to the left.

Notably, for subspaces 1–25 – a regime in which training error decreases continuously – the testing error decreases, attains a minimum, and then begins to increase. Thus, while a 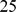 dimensional representation of the original perceptual data is evidently the most accurate achievable with NMF, it is not necessarily the most parsimonious. Inspecting low-order basis vectors, we observed that descriptors with largest-amplitudes were consistent across repetitions of the factorization, and corresponded to broadly applicable labels such as ‘fragrant’, and ‘sickening’ (see Figure 2 for examples). By contrast, higher order basis vectors (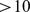) had peak-value descriptors that were highly specific (‘anise’, ‘cinammon’, etc), and somewhat variable between NMF repetitions.

To more quantitatively motivate the choice of subspace size, we applied two techniques commonly used in problems of NMF model selection [23], [26]. First, we plotted reconstruction error (that is, the fraction of unexplained variance) vs subspace size for 250 different repetitions of NMF (Fig. 1C), and compared this to the reconstruction error obtained with PCA performed on the original data (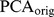) as well as on scrambled data (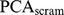) (Fig. 1D) [26]. The slope of 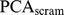 is small and relatively constant for increasing subspace sizes (Fig. 1D), and provides a means for estimating the point after which a given model is explaining noise rather than correlations in data . To visualize this cutoff point, Figure 1D plots the change in variance for each added dimension (differences between successive points in Figure 1C). The reconstruction error rates of both 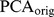 and NMF intersect with 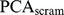 at subspace size 10 (Fig. 1D), indicating that there is no gain in retaining dimensions 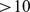 for either dimensionality reduction method. This is consistent with a recently published estimate of the intrinsic dimensionality of this same dataset [11], using PCA. For a further comparison of NMF with PCA, we show cumulative variance plots of PCA and several runs of NMF in Fig. S1.

As a second means for quantifying the intrinsic dimensionality of the Dravnieks data set, we calculated the cophenetic correlation coefficient [23] for several choices of subspace size. Briefly, this method exploits the stochasticity inherent in NMF to determine how reproducible the derived basis set and odor weights are across repetitions of the factorization. Cophenetic correlations 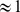 indicate highly reproducible basis sets (see Methods for further explanation). We note that cophenetic correlation analyses can often provide better grounds for excluding certain choices of subspace size that lead to unreliable clustering, rather than privileging a specific number as ‘the’ dimensionality of the data.

The results of our cophenetic correlation analysis are shown in supplementary Fig. S2. Two features are readily apparent: First, there are some notably poor choices of subspace size (such as 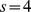 or 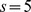). We speculate that the sharp drop at these values is because at these subspace choices, the classification scheme has lost the advantage of being simple and dichotomous, but has yet to support enough categories for accurate and reliable classification. Second, unlike with the reconstruction error criterion (above), there is no monotone decreasing relationship between cophenetic correlation and dimension size that provides an obvious stopping criterion. Our interpretation of this is that there are many good, reduced-dimensionality representations of the Dravnieks data that exhibit sparse structure.

Given that analysis of reconstruction error (Fig. 1D) argues for a choice of 10 dimensions as a cutoff point, and cophenetic correlation analysis suggests there are many well-motivated choices of subspace choices 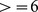 (Fig. S2), we therefore settled on a subspace size of 10 for all further analyses. Visualizations of NMF reconstruction quality for different choices of subspace size are provided in figure S3, which shows that most of the global and local structure of the original data is explained with 10 NMF basis vectors. We wish to note, however, that in general there is no single exact criterion for NMF model selection. There are multiple justifiable choices of subspace size, each of which may lead to different insights about the data, or be useful for different goals.

### Sparseness of basis vectors

An immediate consequence of the non-negativity constraint is sparseness of the basis vectors. As seen in Figure 2, the basis vectors consist of a handful of large values, with the remaining values near or equal to zero. Intuitively, a given basis vector indicates a subset of descriptors that are related and particularly informative (Fig. 2 A), while the set of all basis vectors (Fig. 2B) defines a library of such aggregate descriptors that span the space. Figure 2C shows the first four basis vectors, which have been normalized and ranked in decreasing order to highlight their sparseness. The six most heavily weighted descriptors for each basis vector are shown to the right. Together, these vectors define 4 descriptor axes that can be roughly labeled as ‘fragrant’, ‘woody’, ‘fruity’, and ‘sickening.’ We note that these labels are for purposes of concision only, as each axis is actually a meta-descriptor consisting of a linear combination of more elementary descriptors. A list of rank-ordered descriptors for all 10 dimensions is shown in Table 1.

**Table 1:** 10 largest-valued descriptors for each of the 10 basis vectors obtained from non-negative matrix factorization.

W1	W2	W3	W4	W5	W6	W7	W8	W9	W10
FRAGRANT	WOODY, RESINOUS	FRUITY, OTHER THAN CITRUS	SICKENING	CHEMICAL	MINTY, PEPPERMINT	SWEET	POPCORN	SICKENING	LEMON
FLORAL	MUSTY, EARTHY, MOLDY	SWEET	PUTRID, FOUL, DECAYED	ETHERISH, ANAESTHETIC	COOL, COOLING	VANILLA	BURNT, SMOKY	GARLIC, ONION	FRUITY, CITRUS
PERFUMERY	CEDARWOOD	FRAGRANT	RANCID	MEDICINAL	AROMATIC	FRAGRANT	PEANUT BUTTER	HEAVY	FRAGRANT
SWEET	HERBAL, GREEN, CUT GRASS	AROMATIC	SWEATY	DISINFECTANT, CARBOLIC	ANISE (LICORICE)	AROMATIC	NUTTY (WALNUT ETC)	BURNT, SMOKY	ORANGE
ROSE	FRAGRANT	LIGHT	SOUR, VINEGAR	SHARP, PUNGENT, ACID	FRAGRANT	CHOCOLATE	OILY, FATTY	SULFIDIC	LIGHT
AROMATIC	AROMATIC	PINEAPPLE	SHARP, PUNGENT, ACID	GASOLINE, SOLVENT	MEDICINAL	MALTY	ALMOND	SHARP, PUNGENT, ACID	SWEET
LIGHT	LIGHT	CHERRY (BERRY)	FECAL (LIKE MANURE)	PAINT	SPICY	ALMOND	HEAVY	HOUSEHOLD GAS	COOL, COOLING
COLOGNE	HEAVY	STRAWBERRY	SOUR MILK	CLEANING FLUID	SWEET	CARAMEL	WARM	PUTRID, FOUL, DECAYED	AROMATIC
HERBAL, GREEN, CUT GRASS	SPICY	PERFUMERY	MUSTY, EARTHY, MOLDY	ALCOHOLIC	EUCALIPTUS	LIGHT	MUSTY, EARTHY, MOLDY	SEWER	HERBAL, GREEN, CUT GRASS
VIOLETS	BURNT, SMOKY	BANANA	HEAVY	TURPENTINE (PINE OIL)	CAMPHOR	WARM	WOODY, RESINOUS	BURNT RUBBER	SHARP, PUNGENT, ACID

**Figure 3:**
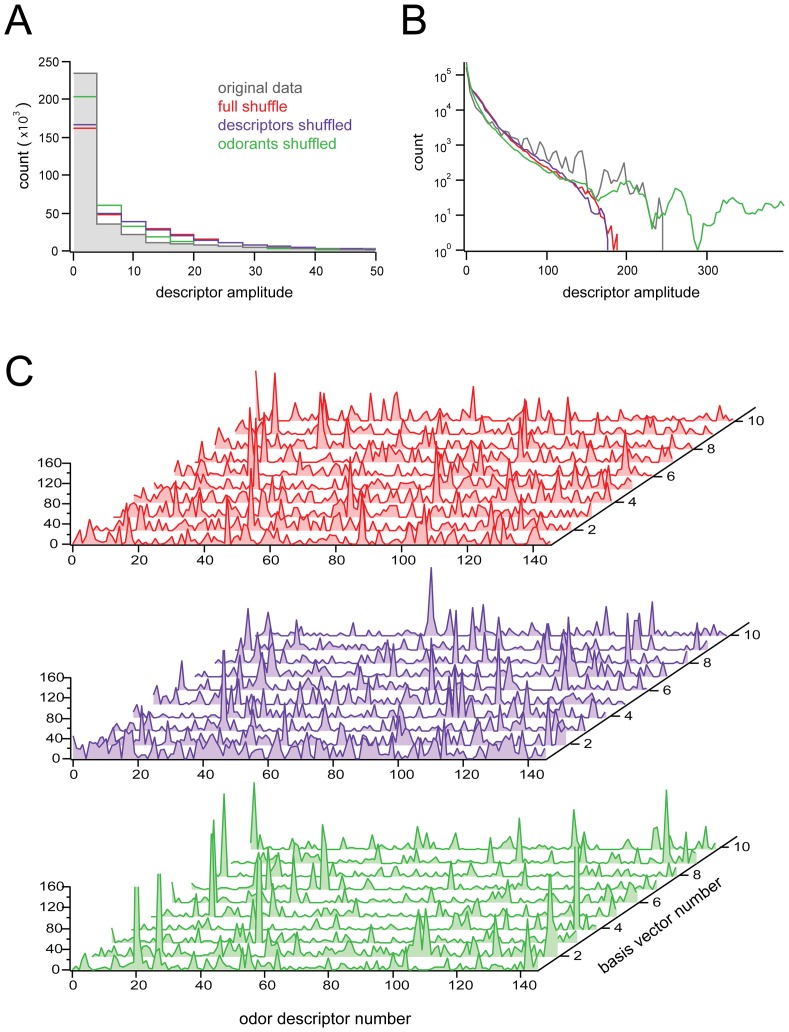
NMF on full, descriptor-only, and odor-only shuffled versions of the data. 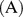
 Peak behavior of histograms obtained from NMF performed on shuffled data, for each of the various shuffling conditions (see text for descriptions). 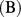 Tail behavior of histograms, same procedure and conditions as in 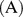; note difference in scaling of axes between 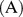 and 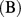. 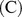 Waterfall plots of basis sets obtained when NMF was applied on shuffled data, for various shuffling conditions. Note the comparative lack of sparseness, relative to the basis set shown in Fig. 3A. Reproducibility of basis vectors across iterations of NMF for shuffled data sets was eliminated, or severely compromised, as shown in Fig. 4.

To ensure that the sparse basis vectors we obtained were not an artifact of the NMF procedure, but rather depended on correlations in the data, we repeated the calculation of W for three shuffled versions of the profiling data (Fig. 3). In the ‘full shuffle’ condition, all elements of the data matrix A were randomly permuted, eliminating all correlations. In the ‘descriptors-shuffled’ conditions, the elements of each column of A were randomly permuted, while in the final ‘odorants-shuffled’ conditions, the elements of each row of A were randomly permuted. In agreement with the idea that the sparseness obtained by NMF is data dependent, sparseness was drastically reduced in the basis sets obtained from all sets of shuffled data (compare Fig. 3C with Fig. 2B).

**Figure 4:**
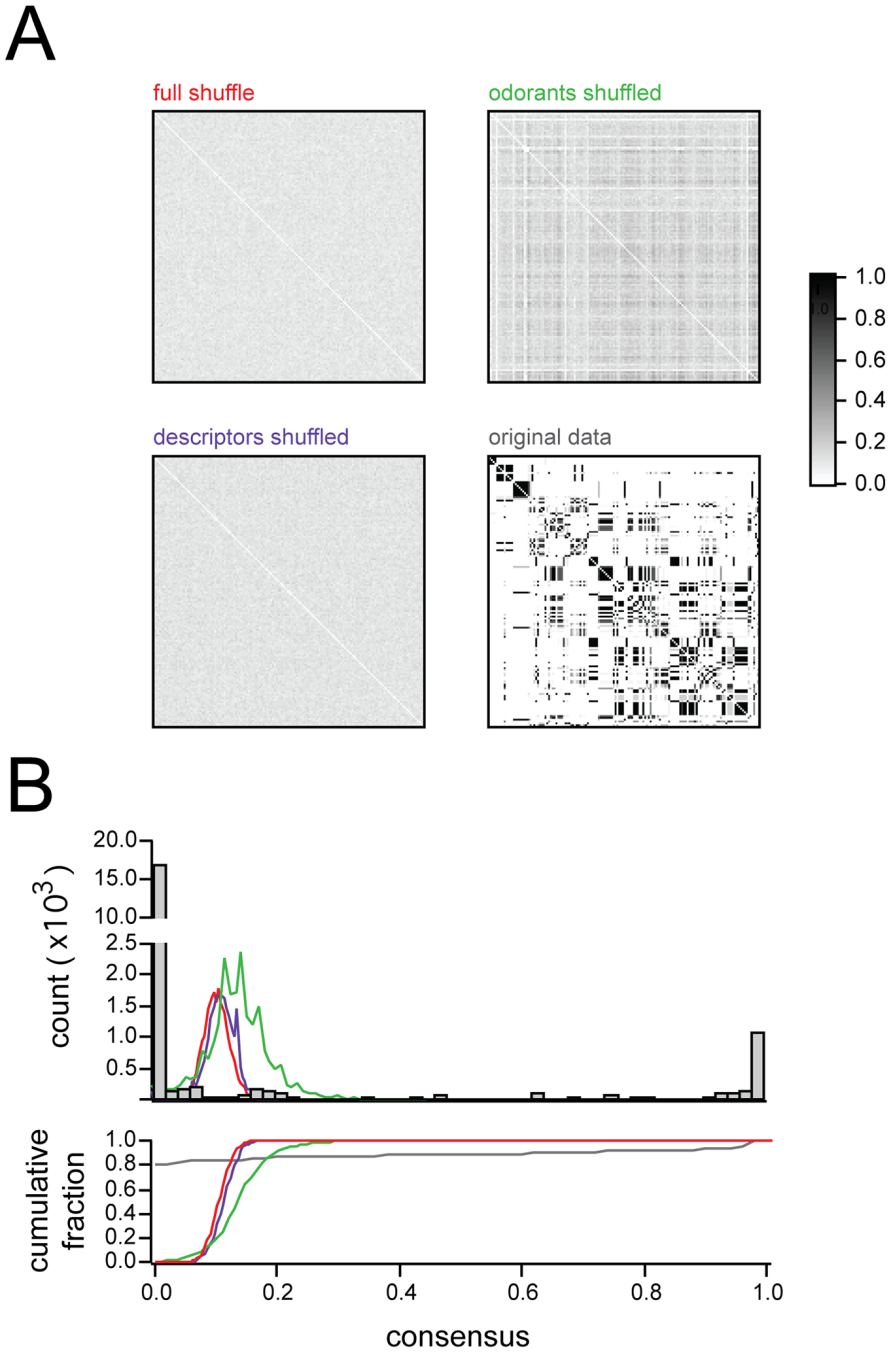
Consensus Matrices for odor-shuffles, descriptor-shuffles, and full-shuffles. 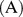
 Consensus matrices (see text) showing reliability of basis sets when NMF is applied to various shuffled versions of the data. Only the original data shows the bimodal distribution of 1s and 0s characteristic of highly reliable clustering. Image ranges and colorscale same for all 4 matrices. 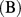 Top: Histograms of consensus matrix values for the three shuffling conditions, and the original data, confirming that only the original data shows a bimodal distribution of 1s and 0s (line colors correspond to labels in 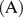). Bottom: Cumulative histograms, same data as above.

In histograms of basis vectors obtained from the full-shuffled and descriptor-shuffled data (Fig. 3A), it was evident that both basis sets contained fewer zero-valued elements than the unshuffled basis set. Interestingly, the long-tail behavior of the histogram was preserved (even enhanced) in the odorants-shuffled condition (Fig. 3B). While this does indicate that a small number of basis vector elements did have very large values in the odorant shuffle cases, this was notably at the expense of peak behavior at zero (Fig. 3A, green). Moreover, basis vectors derived from a given odorant-shuffled matrix were highly inconsistent across repetitions of the factorization, which we assessed by computing consensus matrices (see Methods) documenting the stability of clusters across different iterations of NMF (Figure 4). In brief, we found that only the original data had clusters that were consistent across iterations.

**Figure 5:**
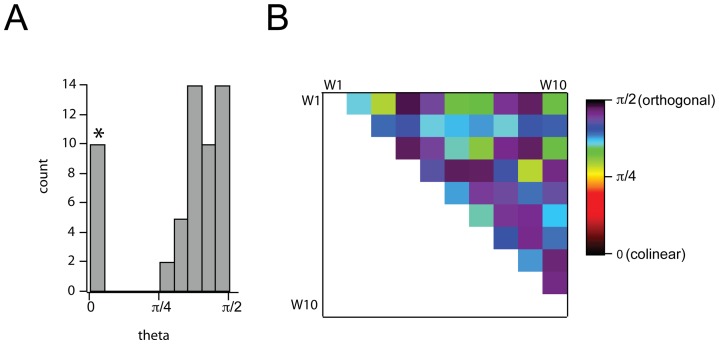
Approximate orthogonality of the NMF basis vectors. 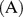
 Histogram of angles subtended by all pairs of basis vectors, 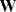. Histogram was constructed for all pairwise comparisons between dimensions, excluding self-comparisons. Bar with (*) denotes self-comparisons. 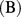 Matrix of pairwise comparisons of angles between dimensions.

While these first several NMF dimensions (Fig. 2, and Table 1) define a perceptual descriptor space reminiscent of that observed previously with PCA, we note that variance is distributed somewhat differently in the NMF vs PCA basis sets. In essence, we have traded degrees of freedom for increased interpretability of individual perceptual dimensions. Interestingly, despite the fact that NMF imposes no formal orthogonality constraint on basis vectors, the perceptual basis set discovered by NMF was still near-orthogonal (Fig. 5); that is, most pairwise comparisons among the basis vectors in 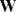 subtend an angle close to 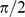 (median angle  = 72.9 degrees).

### Distribution of odors in the new perceptual descriptor space

We next asked how the 144 individual odor profiles (that is, columns of 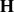) are distributed in the new 10 dimensional perceptual descriptor space spanned by 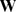. One possibility, for example, is that many of the descriptor space dimensions are redundant, resulting in odors being confined to a thin, low-dimensional slice of the full space. At the other extreme, odors may densely occupy descriptor space, indicating that dimensions contain non-redundant features, with all dimensions necessary to fully characterize odors.

To investigate these and other possibilities, we first examined the structure of 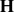, the matrix of odor weights obtained from NMF (recall that each column of 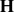 corresponds to an odor, and defines a point in 10-dimensional descriptor space spanned by 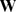; Fig. 1A). We took the Euclidian norm of each column of 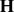, and then sorted all columns into 10 groups defined by their largest coordinate in descriptor space. More explicitly, the 144 columns of 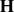 were scanned left to right until one was found with a largest coordinate in dimension 1. This was then assigned as the first column of the re-ordered matrix. The remaining set of columns was similarly scanned, until all columns with a largest first-coordinate had been found. This procedure was then iterated on the remaining dimensions 2–10. Note that this is just a cosmetic reordering of columns that preserves row orderings – no new structure has been added, and no existing structure been destroyed.

**Figure 6:**
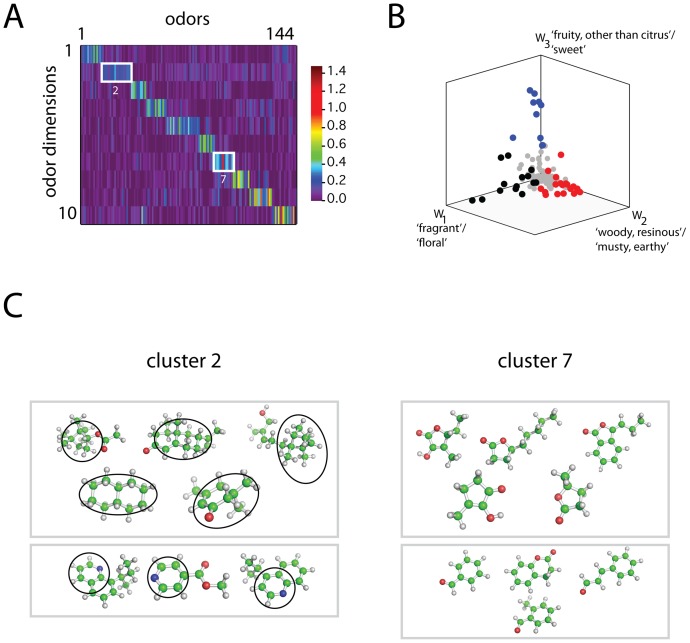
Visualization of odors expressed in coordinates of the new basis. 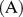
 The weight matrix, 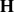, discovered by NMF. Columns of 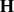 (each column corresponds to a different odor), are normalized and sorted into groups defined by peak coordinate (1–10). 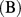 Plot of all 144 odors (each point is a column of 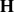) in the space spanned by the first 3 basis vectors, 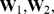 and 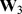. Black, red, and blue points are those with peak coordinates in dimensions 1, 2, and 3 respectively. Gray points are all remaining odors. 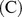 Chemical structures of representative odorants from the second and seventh diagonal blocks of the sorted matrix 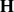 (panel 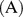).

Intriguingly, this procedure revealed a prominent block diagonal structure to the full matrix 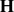 (Fig. 6A) indicating that: 1) a given odor tends to be characterized by a single prominent dimension, and 2) all 10 dimensions are occupied. Furthermore, this suggests that a given odor percept may be considered an instance of one of several fundamental qualities (see discussion).

These two properties can be alternatively visualized when odors (columns of 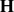) are plotted as points in the 10 dimensional perceptual space spanned by basis set 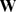. Because this perceptual space is high-dimensional and difficult to represent geometrically, we show a representative 3 dimensional subspace of 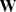. We note that this is not a projection of the data, but rather a selective visualization of a subspace. Figure 6B shows all 144 odors in the space spanned by perceptual dimensions 1–3. Most odors are clustered diffusely near the origin (gray points in Fig. 6B), since their peak coordinates do not reside in this particular 3-D subspace. By contrast, when odors are separated into groups defined by peak coordinate (as in Fig. 6A), it is evident that a given odor tends to be best defined by a single perceptual dimension. The black, red, and blue points in figure 6B, for example, are those points with largest coordinates occurring in the first, second, and third dimensions respectively. While there was notable structural homology among the odors in a given diagonal block of 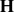 (Fig. 6C), we did not quantify this further in the present work. Figure S4 shows additional representations of odorants distributed in descriptor space, and further highlights the categorical nature of the perceptual space derived from NMF.

**Figure 7:**
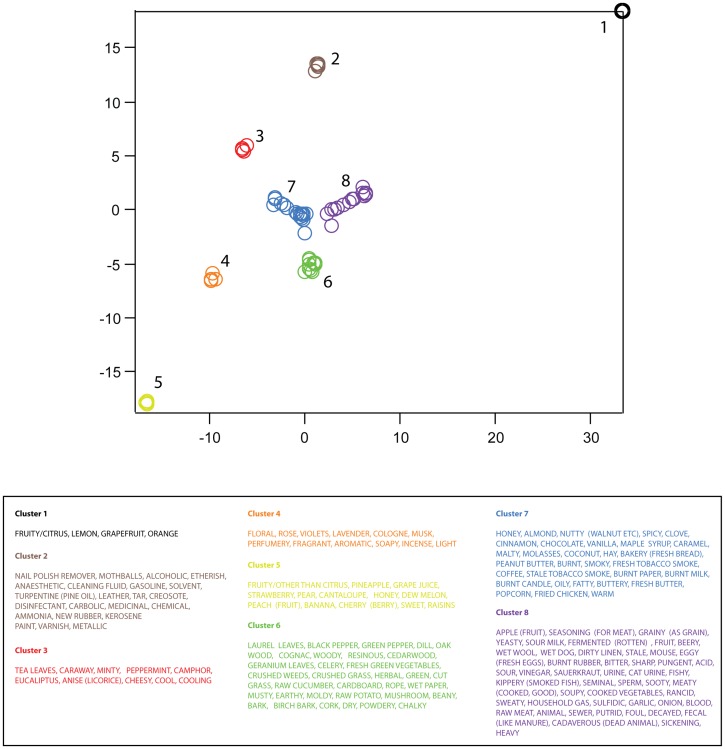
Two-dimensional embedding of the descriptor-space, 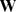. Results of stochastic neighbor embedding (see text) applied to the similarity matrix for 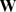. Axis units are arbitrary, but preserve neighbor relations present in the higher dimensional space, 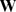. Note that discrete clusters are clearly evident. Clusters were identified by eye, and descriptors composing each cluster are listed in the table below.

**Figure 8:**
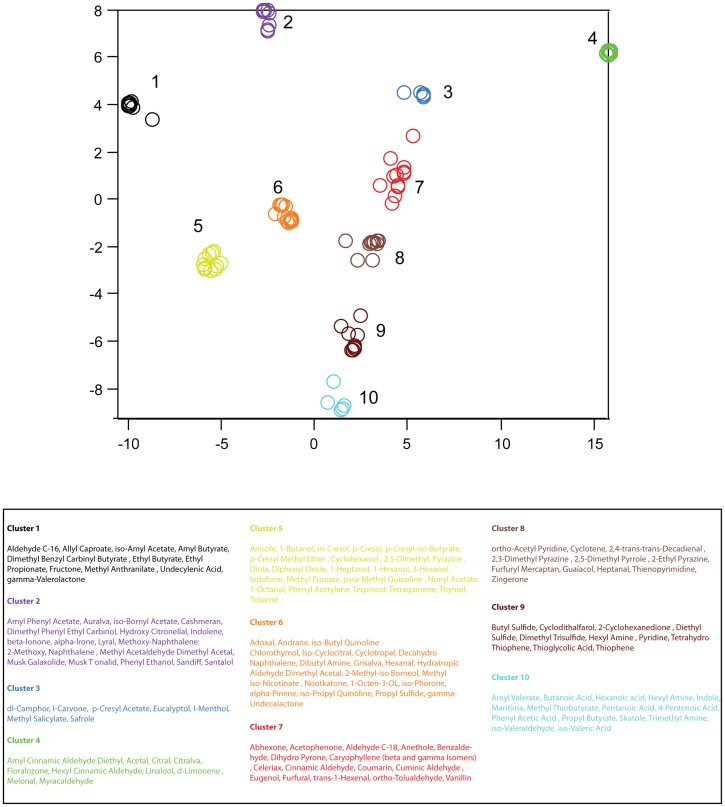
Two-dimensional embedding of the odorant-space, 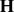. Results of stochastic neighbor embedding (see text) applied to the similarity matrix for 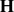. As in figure 7, axis units are arbitrary, but preserve neighbor relationships observed in the full-dimensional space, 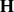. Clusters were identified by eye, and odorants composing each cluster are listed in the table below.

As a final means for investigating whether odorants are smoothly vs. discretely arranged in descriptor space, we constructed two-dimensional embeddings for the matrices 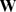 and 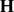 using the stochastic neighbor embedding (SNE) algorithm. Briefly, this technique provides a planar representation of all pairwise distances between odors in the original high dimensional space, such that relative neighbor relations are preserved (e.g. odors that are close together in the original space are also close together in the embedding). Applying SNE to the descriptor space (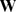), we obtained 8 discrete and non-overlapping clusters of the 146 descriptors, which are shown in Figure 7. Similarly, applying SNE to the space of odorants (H), we obtained 10 discrete and non-overlapping clusters of the 144 odors (Figure 8). In sum, the perceptual descriptor space derived from NMF is not smoothly occupied.

### Bi-clustering of descriptors and odors

The perceptual space, 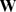, discovered by NMF can be considered a set of 10 meta-descriptors, each of which is a linear combination of more elementary descriptors. While these dimensions are compact and categorical in that a given odor tends to have a prominent single coordinate (Figs. 6 and S4), this may also obscure interesting details about the organization of the descriptor space. For example, within a dimension there may be correlations between specific descriptors and specific odors.

**Figure 9:**
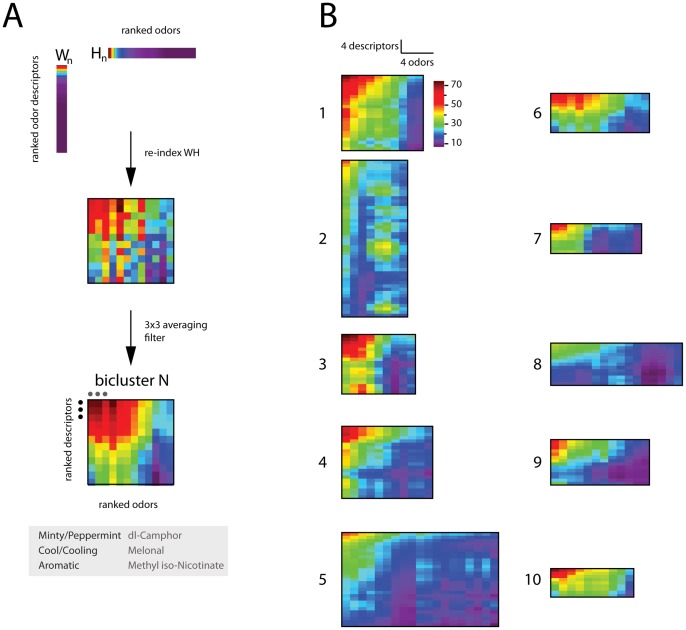
Co-clustering of descriptors and odors. 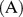
 Overview of method used for defining a bicluster (see text for definition). A column 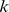 of 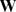 (descriptors), and the corresponding 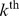 row of 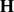 (odors) are rank ordered. The indices derived from the rank-ordering are used to re-order rows and columns of 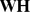 (accomplished by computing the outer product between the rank-ordered 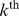 column of 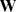 and rank-ordered 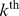 row of 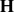), producing a submatrix with high correlation among both odors and descriptors. By the nature of the sorting procedure, these matrices – biclusters – will have their largest values in the upper-left corner. For purposes of visualization, biclusters were convolved with an averaging filter. 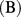 The 10 biclusters defined by NMF on odor perceptual data.

To explore this potential fine-scale structure wherein subsets of odorants show distinct correlations among subsets of descriptors, we sought submatrices of 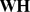 (the NMF approximation to the original data matrix 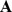 ) with large values in both the descriptor and odorant dimensions (Fig. 9). Briefly, we did this by performing 10-reorderings (one for each perceptual dimension) of rows and columns of 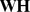 via the process illustrated in figure 9A. Rank-ordering the first column of 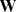, for example, aggregates the peak valued descriptors for the first perceptual dimension, 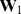. Similarly, rank ordering the first row of H aggregates those odorants with largest weights in 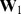. Applying these row and column re-orderings simultaneously to the matrix 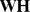 gives a matrix whose largest values are in the upper-left corner.

The clear upper-left organization of these submatrices illustrates that there are sets of odors to which distinct odor descriptors apply. Members of all clusters, as defined by their peak coordinate in the new 10 dimensional descriptor space, are given in Table 2.

**Table 2:** List of compounds in every cluster identified from NMF.

Cluster 1	Cluster 2	Cluster 3	Cluster 4	Cluster 5
1. Isoamylphenylacetate,2. Aurantiol,3. 6,7-dihydro-1,1,2,3,3-pentamethyl-4-(5H)indanone,4. Indol-hydroxycitronellal,5. beta-ionone (low concentration),6. beta-ionone (high concentration),7. N'-[(E)-3-(5-methoxy-2,3-dihydro-1,4-benzodioxin-7-yl) prop-2-enoyl]-2,3-dihydro-1,4-benzodioxine-3-carbohydrazide,8. hydroxyisohexyl 3-cyclohexene carboxaldehyde,9. 2-methoxynaphthalene,10. Diethoxymethane,11. Galaxolide,12. ethylenebrassylate,13. Phenylethyl Alcohol (low concentration)14. Phenylethyl Alcohol (high concentration)	15. Cedrene epoxide,16. bornyl acetate,17. 8-sec-Butylquinoline,18. 2,4,6-trimethylcyclohex-3-ene-1-carbaldehyde,19. decalin,20. dibutylamine,21. Synthetic amber,22. 1,1-Dimethoxy-2-phenylpropane,23. Methyl isonicotinate,24. Nootkatone,25. 1-octen-3-ol,26. isophorone (low concentration),27. isophorone (high concentration),28. Isopropyl quinolone,29. Argeol,30. Gamma-undecalactone,31. 10-undecenoic acid	32. ethylmethylphenylglycidate (low concentration)33. ethylmethylphenylglycidate (high concentration)34. allylcaproate,35. isoamyl acetate,36. n-amyl butyrate,37. Dmbc butyrate,38. ethyl butyrate,39. ethyl propionate,40. Fructone,41. methylanthranilate,42. Pentylvalerate	43. Butyric Acid44. hexanoic acid45. indole46. methylthiolbutyrate47. n-pentanoic acid48. 4-pentenoic acid49.50. . phenylacetic acid51. Propyl butyrate52. Skatole (3-Methyl-1H-indole)53. Isovalerylaldehyde54. isovaleric acid	55. Acetophenone56. Anisole57. 1-Butanol58. 4-cresol59. p-Tolylisobutyrate60. 4-methyl anisole61. cyclohexanol62. 2,5-dimethylpyrazine63. methyl hexyl ether64. 1-hexanol65. 3-hexanol66. iodoform67. methyl furan-3-carboxylate68. 4-methylquinoline69. phenylacetylene70. alpha-terpineol71. 6-methyl-1,2,3,4-tetrahydroquinoline72. Thymol73. Toluene74. 3-Methyl-1H-indole

Cluster 6	Cluster 7	Cluster 8	Cluster 9	Cluster 10
1. Anethole2. 8-sec-Butylquinoline3. carvone4. caryophyllene5. 4-cresyl acetate6. eucalyptol7. Eugenol8. Menthol9. methyl salicylate10. Safrole	11. Abhexon12. Gamma-nonalactone13. Benzaldehyde14. 3,4-dihydrocoumarin15. 3-Propylidene phthalide16. cinnamic aldehyde17. coumarin18. cyclotene19. Furaldehyde20. 2-hexenal21. 2-methylbenzaldehyde22. gamma-valerolactone	23. Vanillin24. 2-acetylpyridine25. 2,4-decadienal26. Pyrazine27. methyl hexyl ether28. 2,5-dimethylpyrrole29. Ethylpyrazine30. Ethylpyrazine31. Heptanal32. n-hexanal33. 1-Octanol34. 2-methyl-5,7-dihydrothieno[3,4-d]pyrimidine	35. Zingherone36. dibutyl sulfide37. Chlorothymol38. 2-Mercaptopropanone39. 1,2-cyclohexanedione40. diethyl sulfide41. dimethyltrisulfide42. furfurylmercaptan43. Guaiacol44. Hexylamine45. Hexylamine46. AC1L18DS	47. polythiophene48. Adoxal49. Amyl cinnamic aldehyde diethyl acetal50. Citral51. Geranonitrile52. Cuminaldehyde53. 4-Methyl-2-(1-phenylethyl)-1,3-dioxolan54. 2-Methyl-4-phenylbutan-2-ol55. phenyl ether56. Floralozone57. Heptanol58. hexylcinnamic aldehyde59. hydroxycitronellal60. linalool61. limonene62. Melonal63. Myrac aldehyde64. n-Nonyl acetate

## Discussion

We have applied non-negative matrix factorization (NMF) to odor profiling data to derive a 10-dimensional descriptor space for human odor percepts. For the data set investigated, individual odor profiles are well-classified by their proximity to a single one of these dimensions, with all 10 dimensions being approximately equally expressed across the set of odors. This is consistent with the notion that olfactory space is high-dimensional [27], and not smoothly occupied [14], [28]. More speculatively, the observation that odors tend to be confined to a single best dimension of the NMF basis (Figure 6, and Figure S4 in supporting information) suggests that a given olfactory percept can be described as an ‘instance’ of one of several fundamental qualities. Whether these proposed qualities are innate or the product of learning is, naturally, an important question, but one that is beyond the scope of this study. In addition, we note two important caveats of the present work. First, the fundamental odor qualities we propose are necessarily provisional, given the limitations of the Dravnieks data set in size and odorant diversity. Second, constraining perceptual judgments to a fixed and possibly limited lexicon (i.e. the 146 descriptors) may obscure the true complexity of odor space.

The perceptual dimensions obtained from NMF identify descriptors that are salient in several previous analyses of odor space [9]–[11], [13], [27], and commonly applied in ratings of odor quality. Moreover, these dimensions are consistent with a broad ecological perspective on olfactory function [29], [30] which emphasizes the importance of chemosensation in coordinating approach, withdrawal, and the procurement of safe food. For example, we observe, as others have, dimensions corresponding to relative pleasantness (‘fragrant’ (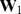), ‘sickening’ (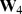)). In addition, most of the remaining dimensions identified appear to correspond to cues of potential palatability/nonpalatability: ‘fruity, non-citrus’ (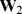), ‘woody, resinous’ (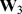), ‘chemical’ (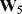), ‘sweet’ (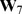), and ‘lemon’ (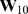). We hasten to note that the labels applied above are only an aid to intuition, as each perceptual basis is really a meta-descriptor consisting of linear combinations of more elementary descriptors. Moreover, it is possible that such linear combinations obscure interesting details about the exact positions of these more elementary descriptors. For a thorough treatment of this issue, one should consult Zarzo et al [9], [31].

While several of these same principal qualities have been identified before, NMF describes a notably different representation of the space in which they reside. Specifically, NMF leads to a description of odor space defined by dimensions that apply categorically. By contrast, odors in PCA space are more diffusely distributed across dimensions. Moreover, odors in PCA space (as well as spaces derived from multidimensional scaling and factor analysis) tend to be smoothly distributed in subspaces that span multiple axes, though heirarchical applications of PCA have identified several quality-specific clusters [9]. Naturally, these differences in the representation of odor space are a consequence of the different constraints applied when obtaining a basis from PCA vs NMF. Whereas PCA basis vectors are chosen to be orthogonal, and allow any linear combination of variables, NMF basis vectors are constrained to be non-negative, allowing only positive combinations of variables. It is worth noting, however, that the NMF basis set is still approximately orthogonal (mean pairwise angle between different basis vectors is 72.9 degrees (Fig. 5)). Moreover, NMF is capturing structure in the data beyond simple first-order statistics, as applying NMF to scrambled versions of 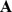 fails to produce sparse and perceptually meaningful basis vectors ( Fig. 3).

Intuitively, the non-negativity constraint produces NMF basis vectors defined by subsets of descriptors that are weighted and co-applied in particularly informative combinations, defining dimensions that range from absence to presence of a positive quantity. This contrasts to basis vectors and dimensions derived from other techniques, which extend from one quality to that quality's presumed opposite. Such dimensions have intuitive interpretations in some cases, for example, the experimentally supported ‘pleasantness’ dimension corresponding to principal component 1 (PC1), which ranges from ‘fragrant’ to ‘sickening’. Interestingly, constraining the NMF subspace to 2 shows that most odors fall homogeneously along a continuum reminiscent of the first principal component (Fig. S5 in supporting information). However, second and higher order PCs become progressively more difficult to interpret, spanning such qualities as ‘woody, resinous’ 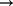 ‘minty, peppermint’ (PC2), and ‘floral’ 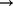 ‘spicy’ (PC3). Whether odor percepts are more accurately represented as residing in dimensions that span oppositely valenced qualities, or dimensions that represent only a single quality will depend on whether there is systematic opponency in peripheral or central odor representations.

It may be possible to observe physiological properties of odor representations indicative of one kind of representation vs. another. If the underlying perceptual dimensions of odor space are categorical, one would expect relative similarity between odor representations for odors occupying the same putative perceptual dimension. Similarly, one would expect abrupt, state-like transitions in neural representations of slowly morphing binary mixture stimuli whose component odors nominally ‘belong’ to different perceptual dimensions. Consistent with these criteria, a recent study has shown discrete transitions in the ensemble activity of the zebrafish olfactory bulb during such odor morphs ([14], but see [32]).

Our study has some limitations that should be noted. Chief among these is the small size of the odor profiling data set used relative to the much larger set of possible odors, which may limit the generality of our findings. In future studies, it will be necessary to extend the NMF framework to larger sets of odors than the 144 investigated presently, such that a more complete and representative sample from odor space is obtained. Another limitation pertains to the ‘subjective’ nature of odor profiling data. While profiles are quantitative in the sense that they are stable and reliable across raters [33], it is clearly important to corroborate profiling-derived estimates of the intrinsic dimensionality of odor space, as well as proposals for how this space is structured, with psychophysical tests of discriminability [34]. It would be interesting, for example, to test whether the approximately orthogonal axes we observe are recapitulated in data derived from tests of pairwise discriminability. Finally, our analysis cannot distinguish between perceptual vs. cognitive influences on the organization of human odor space. One possibility is that the coarse division of odor-space into quality-specific axes reflects the existence of fixed points or attractors [14], [28] that guide odor processing dynamics; similarly, there may exist a set of especially stable, prototypical glomerular maps that serve a related functional role. Another possibility is that early olfactory processing only resolves odor quality to a degree sufficient to rank relative pleasantness, with further parsing of this percept into discrete categories occurring through mechanisms involving learning and context.

In summary, we have shown that olfactory perceptual space can be spanned by a set of near-orthogonal axes that each represent a single, positive-valued odor quality. Odors cluster predominantly along these axes, motivating the interpretation that odor space is organized by a relatively large number of independent qualities that apply categorically. Independently of whether our description of odor space identifies innate or ‘natural’ axes determined by receptor specificities, it provides a compact description of salient, near-orthogonal odor qualities, as well as a principled means for identifying and rating odor quality. Finally, our study has identified perceptual clusters that may help elucidate a structure-percept mapping.

## Supporting Information

Figure S1 **Comparison of PCA and NMF.** Plot of cumulative fraction of variance explained for PCA and NMF, for various choices of subspace size.
(TIF)

Figure S2 **Cophenetic correlation vs. choice of subspace size.** Cophenetic correlation obtained for NMF representations of increasing subspace size. Procedure is defined in the text.
(TIF)

Figure S3
**NMF-derived approximations of odor profiles** 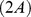 Image of original data (left) and NMF-derived approximations 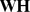 for subspaces of 5 (center) and 10 (right). Same range and color scale for all images. Because the data matrix contains many small and zero-valued entries among sparse, large-valued entries, the colorscale has been gamma-transformed (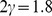) for better visualization and comparisons. Arrowheads indicate columns shown in more detail in panel below. 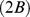 Detailed representation of columns 70–74 of original data matrix 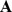 (black traces) and NMF approximations to those columns by 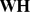 for a 10 dimensional subspace (red traces).(TIF)

Figure S4
**Representations of odorants distributed in perceptual space.** 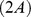 Star plots of odorants (columns of 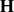). Odorant weight vectors are wrapped on 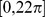 for visualization purposes. Left: three example odorants and their distributions in perceptual space, showing that a given odorant tends to occupy a single one of ten perceptual dimensions, to the exclusion of others. Right: star plot of all 144 odorants in the perceptual space. Colors indicate odors with a common peak coordinate in the 10-D descriptor space. 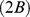 Visualizations of various three-dimensional subspaces of the matrix 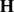, as in Figure 6.(TIF)

Figure S5
**NMF reveals hedonic valence of odors.** For a choice of subspace 2, NMF reveals the hedonic valence of odors. 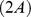 left column: basis vectors returned for NMF with subspace 2. right column: normalized amplitudes and descriptors for leading values of rank-ordered basis vectors. 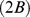 Plot of all 144 odors in the space spanned by 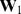, 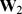 (analogous to plots shown in Fig. 6 in the main manuscript). Colors indicate classification based on largest coordinate (black, 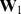, gray, 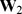), showing coarse categorization into good-vs-bad smelling odors.(TIF)
