# Supplementary figures and images for: Categorical Dimensions of Human Odor Descriptor Space Revealed by Non-Negative Matrix Factorization
Source: PLoS One. 2013 Sep 18;8(9):e73289. doi: 10.1371/journal.pone.0073289 (PMC3776812; doi:10.1371/journal.pone.0073289)

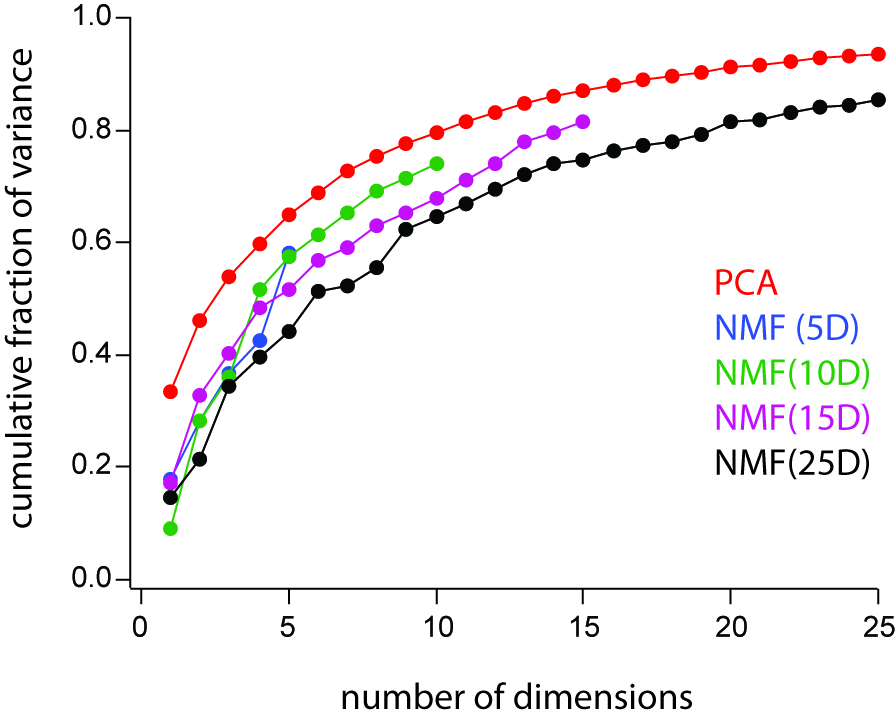

Supplement: Figure S1 — Comparison of PCA and NMF. Plot of cumulative fraction of variance explained for PCA and NMF, for various choices of subspace size. (TIF) [file pone.0073289.s001.tif]

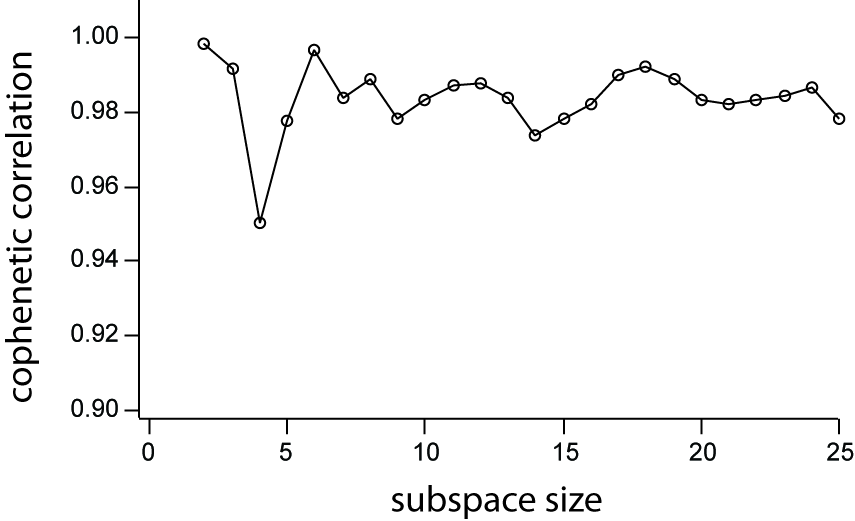

Supplement: Figure S2 — Cophenetic correlation vs. choice of subspace size. Cophenetic correlation obtained for NMF representations of increasing subspace size. Procedure is defined in the text. (TIF) [file pone.0073289.s002.tif]

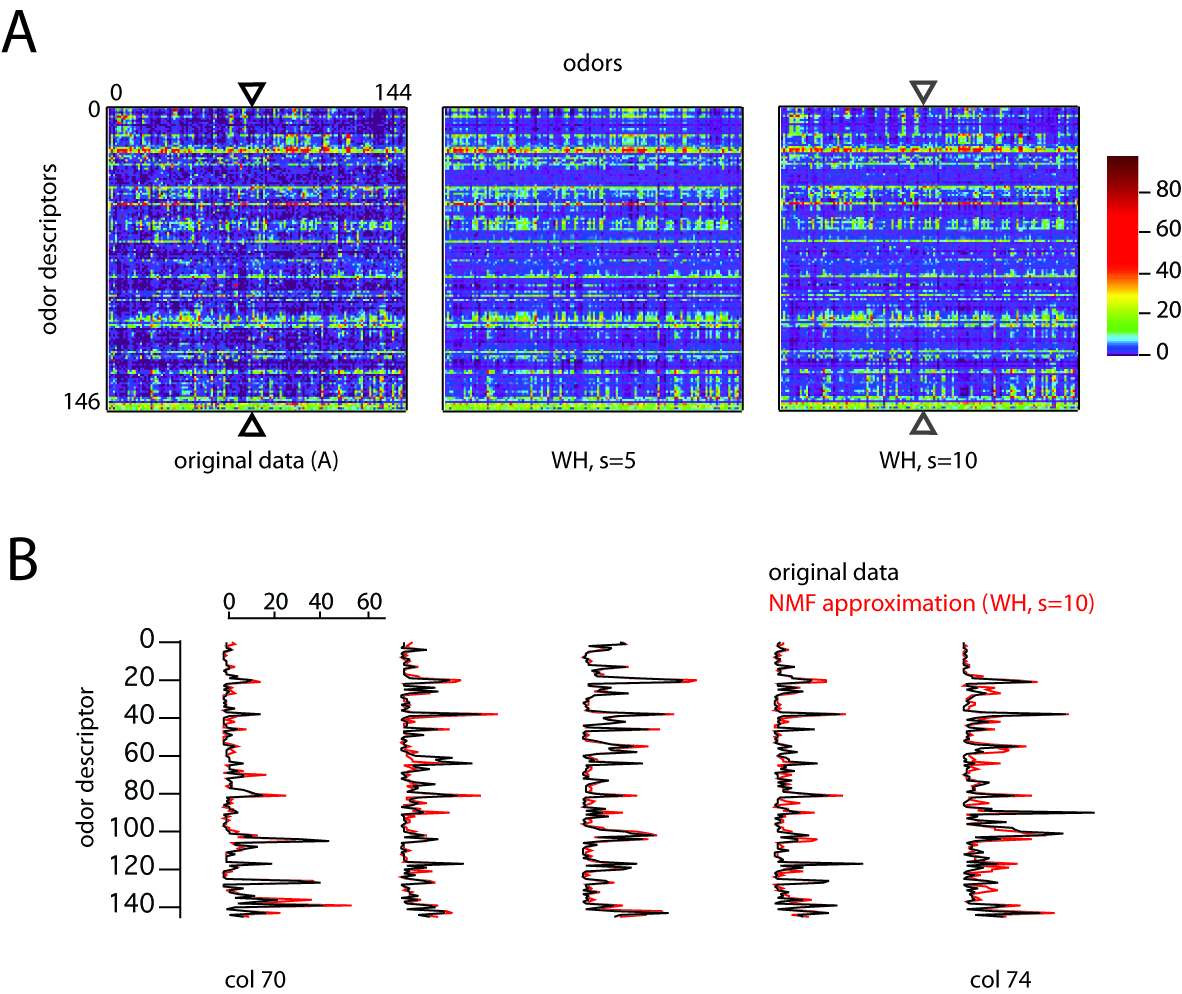

Supplement: Figure S3 — NMF-derived approximations of odor profiles Image of original data (left) and NMF-derived approximations for subspaces of 5 (center) and 10 (right). Same range and color scale for all images. Because the data matrix contains many small and zero-valued entries among sparse, large-valued entries, the colorscale has been gamma-transformed () for better visualization and comparisons. Arrowheads indicate columns shown in more detail in panel below. Detailed representation of columns 70–74 of original data matrix (black traces) and NMF approximations to those columns by for a 10 dimensional subspace (red traces). (TIF) [file pone.0073289.s003.tif]

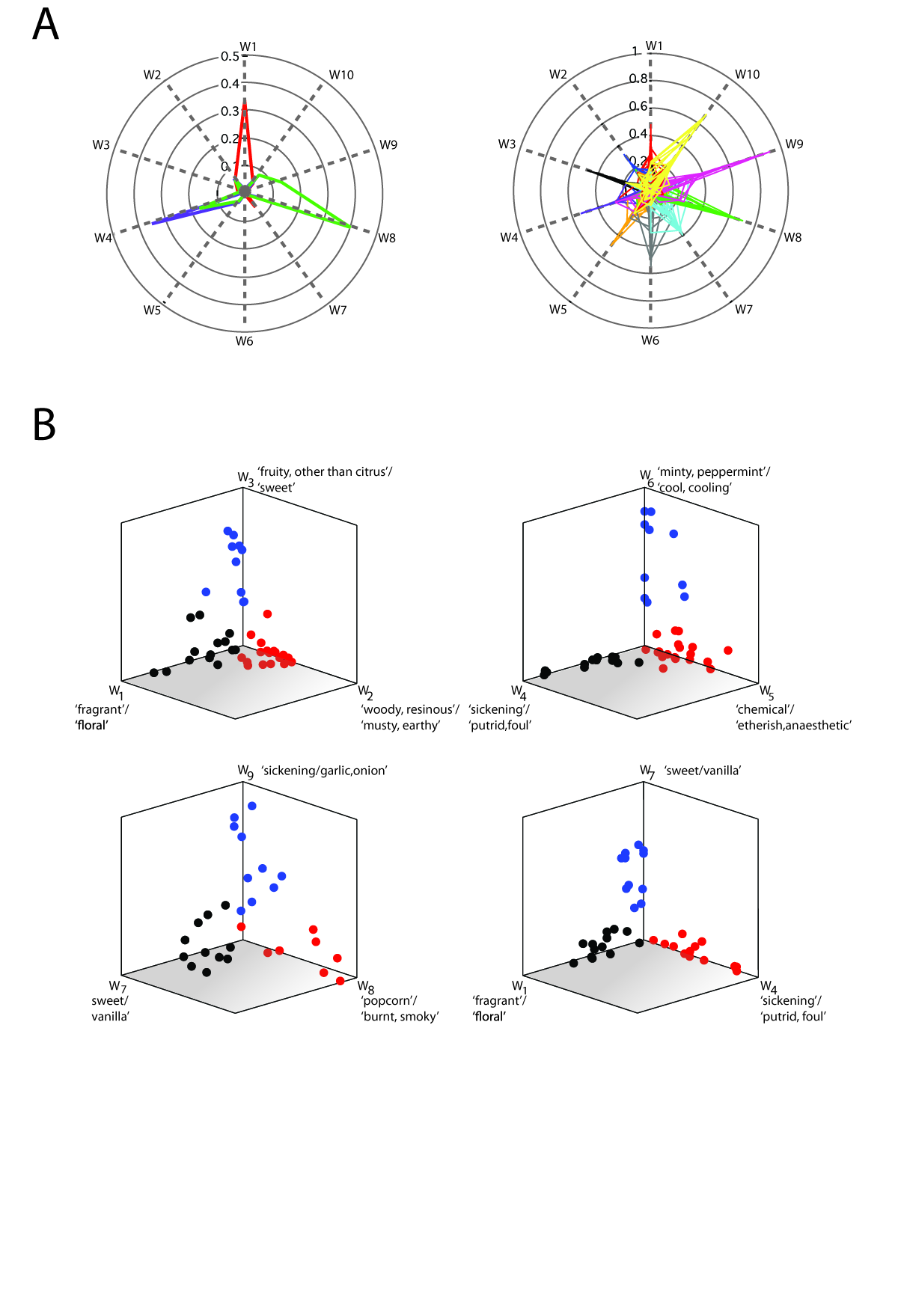

Supplement: Figure S4 — Representations of odorants distributed in perceptual space. Star plots of odorants (columns of ). Odorant weight vectors are wrapped on for visualization purposes. Left: three example odorants and their distributions in perceptual space, showing that a given odorant tends to occupy a single one of ten perceptual dimensions, to the exclusion of others. Right: star plot of all 144 odorants in the perceptual space. Colors indicate odors with a common peak coordinate in the 10-D descriptor space. Visualizations of various three-dimensional subspaces of the matrix , as in Figure 6. (TIF) [file pone.0073289.s004.tif]

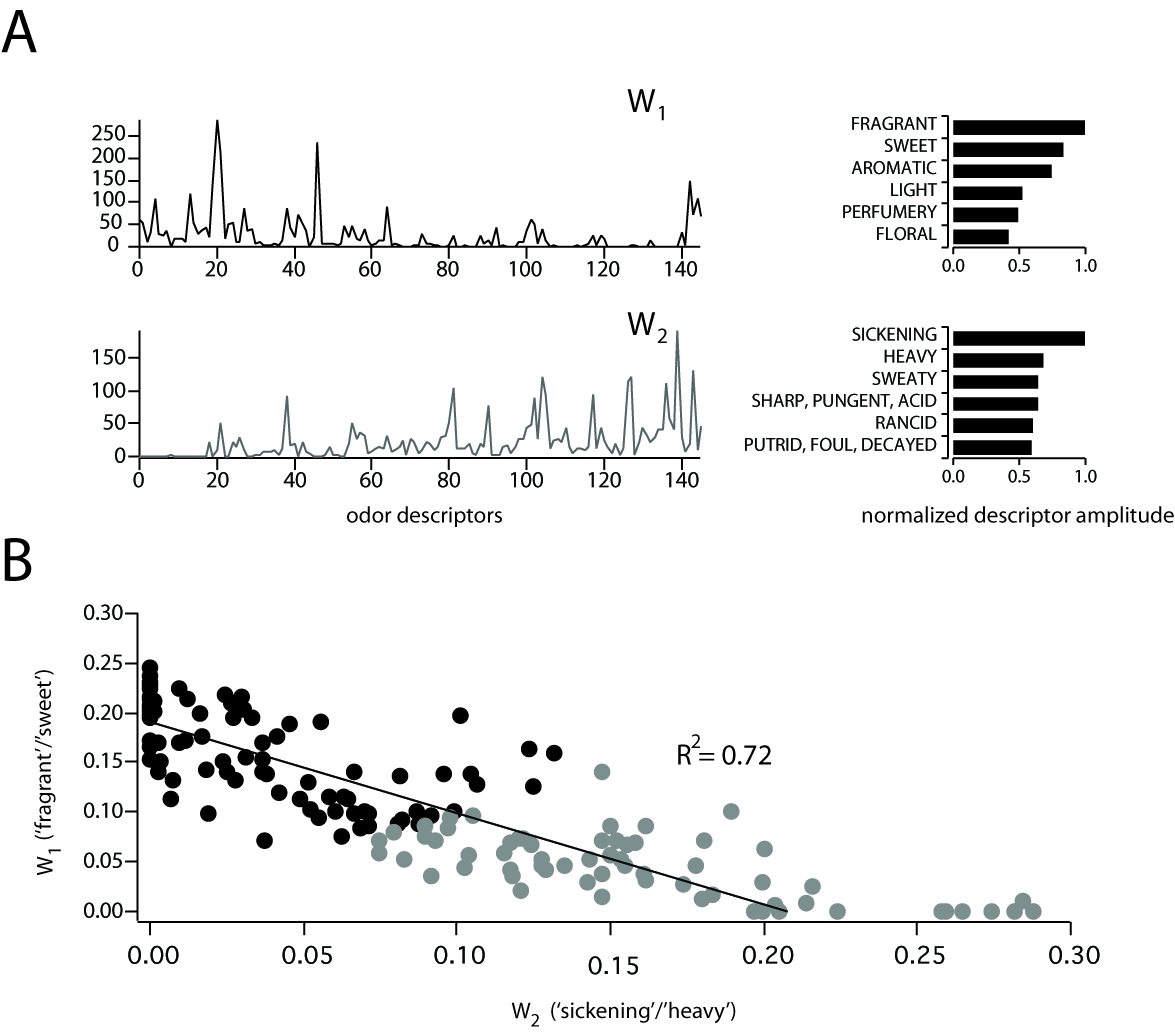

Supplement: Figure S5 — NMF reveals hedonic valence of odors. For a choice of subspace 2, NMF reveals the hedonic valence of odors. left column: basis vectors returned for NMF with subspace 2. right column: normalized amplitudes and descriptors for leading values of rank-ordered basis vectors. Plot of all 144 odors in the space spanned by , (analogous to plots shown in Fig. 6 in the main manuscript). Colors indicate classification based on largest coordinate (black, , gray, ), showing coarse categorization into good-vs-bad smelling odors. (TIF) [file pone.0073289.s005.tif]
